# Characterization of the mitochondrial genome of *Phyllomimus* sp. (Orthoptera: Pseudophyllidae)

**DOI:** 10.1080/23802359.2017.1413296

**Published:** 2017-12-07

**Authors:** Zhong-Ying Qiu, Hao Yuan, Xiaolong Wang, Yuanyuan Cui, Ting Lian, Shaoli Mao

**Affiliations:** a School of Basic Medical Sciences, Xi’an Medical University, Xi’an, China;; b School of Life Sciences, Shaanxi Normal University, Xi’an, China;; c Research Center for Prevention and Treatment of Respiratory Disease, School of Clinical Medicine, Xi’an Medical University, Xi’an, China;; d Xi’an Botanical Garden of Shaanxi Province, Xi’an, Shaanxi, China

**Keywords:** Mitogenome, phyllomimus sp, pseudophyllinae, phylogeny

## Abstract

The nearly complete mitochondrial genome (mitogenome) without *tRNA-Met* and with a partial A + T-rich region of *phyllomimus* sp. has been sequenced with the length of 15,543 bp. We found 140 bp-long intergenic spacers (IGSs) which located between *tRNA-Gln* and *ND2*, while this region should be *tRNA-Met* in most orthopteran mitogenomes. The content of As, Ts, Cs Gs and AT in the mitogenome is 37.3%, 32.5%, 20.6%, 9.5% and 69.8%, respectively. All protein-coding genes start with typical ATN codon except for *ND1*, which initiates with TTG codon instead, and end with either complete TAA/TAG codons or incomplete T(aa) codons. Phylogenetic analysis indicated that genetic distances of *phyllomimus* sp. and *Orophyllus montanus* was closer than other species.

Pseudophyllinae consists about 260 genera and 1000 species, distributed in tropical and subtropical areas. Only *Orophyllus montanus* (KT345951) and *Phyllomimus detersus* (NC_028158) mitogenomes have been reported in the Genbank so far. In this study, we sequenced and analysed the mitogenome of *phyllomimus* sp., with the number of GenBank MG366126. The specimen of *phyllomimus* sp. was collected from Xing’an County (Guangxi, China) in 2009, and was deposited in Molecular and Evolutionary Lab in Shaanxi Normal University in China. The total genomic DNA was isolated from the leg muscle of the sample using the phenol–chloroform extraction method and the orthopteran universal mitochondrial primers were performed following the study of Liu et al. (2006). We used the Staden Package 1.7 to assemble and annotate the mitogenome of *phyllomimus* sp. (Staden et al. 2000).

The mitogenome of *phyllomimus* sp. is at least 15,543 bp in length, including 13 protein-coding genes, 21 tRNA genes, two rRNA genes and one incomplete A + T-rich region, lacking of the *tRNA-Met* and partial A + T-rich region. But we found a 140 bp-long intergenic spacers, which located between *tRNA-Gln* and *ND2*, while this region should be *tRNA-Met* in most orthopteran mitogenomes.

 The tRNA genes were predicted by the online software tRNAScan-SE (Lowe and Eddy 1997), and the length ranked from 62 bp (*tRNA-Ala*) to 71 bp (*tRNA-Val* and *tRNA-Lys*). There are a total of 42 bp overlaps in the mitogenome of *phyllomimus* sp. at 10 locations with the length ranging from 1 bp to 8 bp, and the longest overlaps occurs between *tRNA-Trp* and *tRNA-Cys* and between *tRNA-Tyr* and *COX1*. Total 399 bp were found in 17 intergenic spacers in this mitogenome with the length ranging from 2 to 140 bp, and the longest one was 140 bp between *tRNA-Gln* and *ND2*. All tRNAs could be folded into typical cloverleaf secondary structures, except *tRNA-Ser* (AGN), whose dihydrouridine arm forms a simple loop as in most other insects. The length of *12S rRNA* were 823 bp and *16S rRNA* was 1580 bp, located between *tRNA-Leu*(CUN) and A + T-rich region. The typical ATN start codon was presented in 12 PCGs, except *ND1* whose start codon was TTG. Most PCGs of *phyllomimus* sp. end with TAA, while *COX3* ends with TAG and *ND4* and *COX2* ends with the incomplete stop codon TA and T, respectively. The sequenced partial A + T-rich region was 119 bp in length, located between *12S rRNA* and *tRNA-Ile*. In other Ensiferan species, the length of the A + T-rich region ranges from 70 bp (*Ruspolia dubia*) (Zhou et al. 2007) to 3119 bp (*S. longifissa*) (Liu et al. 2013).

 To further validate the mitogenome of *phyllomimus* sp., a phylogenetic analysis was built in the superfamily Tettigonioidea based on 13 PCGs, 2 rRNA and 9 tRNA genes (not including *tRNA-Ile*, *tRNA-Gln* and *tRNA-Met*) with 26 ingroup and one outgroup (Figure 1). The result shows that *phyllomimus* sp. can be unambiguously grouped in pseudophyllinae with high bootstrap value supporting. *Phyllomimus* sp. and *Orophyllus montanus* are the sister group.

**Figure 1.**
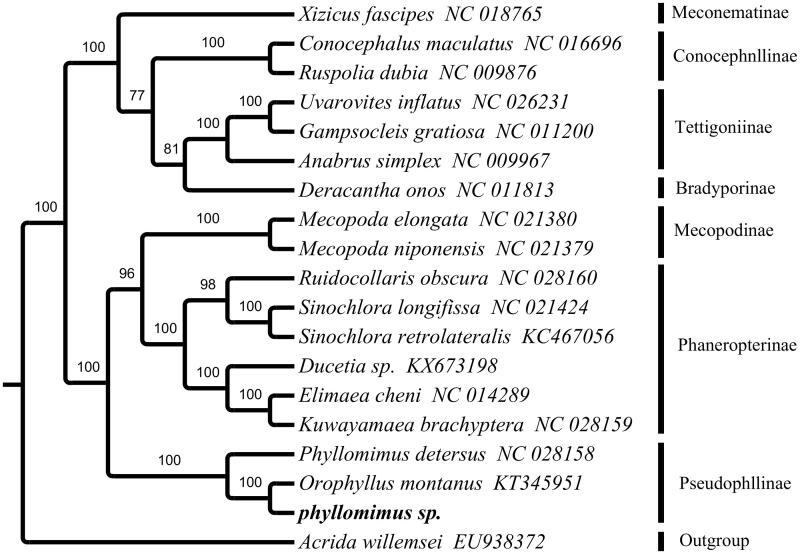
The maximum-likelihood tree of phyllomimus sp. and other 18 species including 17 close-related species and one outgroup species based on mitochondrial PCGs and rRNAs concatenated data.
